# Day-ahead optimal dispatch considering demand response compensation and carbon trading under uncertain environment

**DOI:** 10.1371/journal.pone.0324470

**Published:** 2025-06-09

**Authors:** Ze Ye, Deping Liang, Meihui Wang, Lei Chen

**Affiliations:** 1 School of Economics and Management, Changsha University of Science and Technology, Changsha, China; Aalto University, FINLAND

## Abstract

To fully explore the regulation resources on both sides of the source and load under uncertain environment and collaboratively achieve the energy saving and emission reduction goals, a low-carbon economic optimization dispatch model combining demand response and carbon trading mechanism is proposed in this paper. Firstly, the economic principle of demand response (DR) is analyzed, as well as the demand response compensation model is constructed for shiftable loads and curtailable loads respectively. Second, we describe the source-load synergistic low-carbon effect. The source side further reduces carbon emissions by establishing a reward-punishment laddered carbon trading model. Accordingly, the optimization model is constructed with the objective of minimizing the sum of DR compensation cost, carbon trading cost and system operation cost. The triangular fuzzy method is used to deal with the uncertainty problem of new energy and load forecasting. Finally, the economic and low-carbon nature of this proposed model is verified by simulation and example analysis.

## 1 Introduction

Since the Industrial Revolution, the exponential growth of the global economy has driven a super-linear increase in electricity demand. However, traditional fossil fuel-based power generation has not only accelerated the depletion and scarcity of fossil fuel reserves but also intensified global warming and environmental pollution. As a result, the pursuit of clean and renewable energy sources to replace conventional power generation has become a critical priority for countries around the world [1]. As the world’s largest electricity consumer, China proposed its “dual-carbon” goals in 2020, aiming to reach peak carbon emissions by 2030 and achieve carbon neutrality by 2060. This commitment underscores the determination of major power markets to transition toward sustainable and environmentally responsible energy systems [2,3]. Within this transformative context, it is increasingly important to develop a clean, low-carbon, and economically feasible integrated energy system that features energy complementarity, integrated energy supply, and source-load synergistic.

To promote energy conservation and emission reduction, countries worldwide have actively developed their own carbon trading systems. The European Union (EU), the United States (US), and China launched their respective carbon markets in 2005, 2009, and 2021, with the power generation sector being the first to be included. As a crucial mechanism for reducing carbon emissions and enhancing the output potential of renewable energy, carbon trading systems provide both economic incentives and regulatory constraints to achieve emission reductions. Qian et al. [4] investigated the optimization of power dispatch after incorporating wind energy into the carbon trading framework. Their study found that higher carbon prices reduce wind power curtailment and promote the adoption of cleaner energy sources, although the associated increase in operating costs can weaken incentives for the power sector to implement such changes. Wang et al. [5] designed a carbon credit trading mechanism integrating electricity-carbon market coupling models and profit realization frameworks for market participants, further elucidating correlations among carbon credits, carbon pricing, renewable energy consumption ratios, and associated costs. Building on asymmetric Nash bargaining theory, Chen et al. [6] developed a cooperative carbon trading mechanism optimized for multi-park integrated energy systems. In parallel, Lu et al. [7] proposed a peer-to-peer (P2P) joint electricity-carbon (E&C) trading model accommodating distribution network transaction preferences to co-optimize energy and carbon permit trading. Liu et al. [8] analyzed carbon trading policies’ impact on total-factor carbon productivity, revealing their dual role in fostering technological advancement, factor accumulation, scale optimization, and energy substitution—simultaneously promoting economic growth and emission reduction. Zhang et al. [9] employed system dynamics modeling to unravel interdependencies among carbon markets, tradable green certificate systems, and electricity markets, exposing complex interactions between carbon prices, renewable certificate prices, and electricity tariffs under varying coupling mechanisms. Zhao et al. [10] constructed a dynamic recursive computable general equilibrium model to quantify the synergistic effects of carbon trading and electricity market reforms on energy, economic, and environmental dimensions, identifying policy synergies in industrial structure optimization, energy mix transition, and carbon mitigation. Previous studies have considered the cost of carbon emissions, which contributes positively to energy conservation and emission reduction in power systems. However, they have not segmented carbon emissions into specific intervals.

The implementation of carbon trading mechanisms has demonstrated significant potential in promoting renewable energy consumption [11,12]. However, the increasing grid integration of intermittent renewable energy sources exacerbates system operational uncertainties. To ensure the reliability and economic viability of dispatch strategies, it is imperative to account for uncertainties in renewable generation output and load demand when formulating system optimization models [13]. Various methods have been proposed to address uncertainty in this context. Stochastic Optimization (SO) depends on assumed probability distributions but requires extensive scenario sampling and incurs high computational complexity [14]. Robust Optimization (RO) prioritizes worst-case scenarios, often sacrificing economic efficiency due to excessive conservatism [15]. Interval Optimization (IO) can describe uncertainty bounds but struggles to quantify risk probabilities [16]. Although Information Gap Decision Theory (IGDT) and Distributionally Robust Optimization (DRO) partially overcome these limitations, they still suffer from drawbacks such as complex fuzzy set assumptions and high sensitivity to data [17–19]. Given these methodological shortcomings, fuzzy optimization emerges as a compelling alternative, exhibiting unique advantages under conditions of incomplete or partially known information. While Cui et al. [20] and Qiu et al. [21]successfully employed fuzzy methods to balance risk-cost tradeoffs in data-scarce environments, their studies did not account for the low-carbon potential of source-side demand response.

Demand response is progressively acknowledged as a pivotal flexibility resource for advancing low-carbon transitions on the demand-side of power systems [22]. By leveraging price signals to incentivize flexible load participation in optimal dispatch, DR demonstrates dual decarbonization pathways: reducing peak-time reliance on carbon-intensive generation while enhancing renewable energy accommodation efficiency. Numerous studies have proposed DR integration into system dispatch to guide demand-side flexibility resources in adjusting electricity usage, achieving peak shaving and valley filling while promoting renewable energy utilization [23–26]. Mansouri et al. [27] and Yang et al. [28] employed multi-objective optimization models to analyze consumer participation in flexible electricity purchasing through DR. Their findings indicate that DR implementation reduces system operating costs, increases renewable energy consumption, and decreases carbon emissions, thus achieving both economic and environmental benefits. Aalami et al. [29] developed an economic model based on price elasticity of demand and customer benefit functions, targeting two DR scenarios—interruptible/consumable and capacity market—to enhance load profiles and maintain customer satisfaction. Yu et al. [30] proposed a real-time pricing method for implementing a price-based DR scheme in Iran. Their seasonal analysis demonstrated the positive impact of DR on technical, economic, and environmental performance in grid-connected microgrids. Pratik et al. [31] integrated an incentive-based DR scheme with network reconfiguration in microgrids, incorporating consumer discomfort costs from load shedding into the objective function. Bishwajit et al. [32] formulated incentive-driven DR models to quantify customer elasticity, identifying optimal incentive thresholds for 40% participation rates through sensitivity analyses. Martina et al. [33] devised a bi-level optimization structure coordinating production scheduling with district heating demand management, employing genetic algorithms for upper-level DR optimization and linear programming for lower-level production planning. Zeng et al. [34] constructed natural gas DR models based on dynamic pricing theory, integrating supplier-consumer motivations and price elasticity mechanisms. Ren et al. [35] incorporated five types of extreme natural disasters to model the resilience of integrated energy systems under high-impact events, combining price-based DR scenarios and accounting for uncertainty. Their results indicate that resilient DR optimization can maintain load supply during such events. However, existing DR models primarily consider curtailable or shiftable loads in isolation, lacking detailed classification of flexible load types. Moreover, current research often emphasizes DR’s peak shaving and valley filling capabilities, while the potential of the source-load synergistic low carbon mechanism remains underexplored.

Existing studies have established a foundational understanding of carbon trading mechanisms and DR applications in power systems. As systematically summarized in Table 1, current research exhibits four critical limitations:

**Table 1:** Comparison of the literature review with our proposed method.

Refs.	Carbon trading			DR		Uncertainty			Emission
	Cost	Ladder	Reward and Punishment	Shiftable	Curtailable	Load	Renewable	Treatment	
[20]	✓	✓	✘	✘	✘	✓	✓	Trapezoidal Fuzzy	✓
[36]	✓	✘	✘	✓	✓	✓	✓	RO	✓
[37]	✓	✓	✘	✓	✓	✘	✘	–	✓
[38]	✓	✓	✓	✓	✘	✘	✘	–	✓
[39]	✘	✘	✘	✓	✘	✓	✓	RO	✘
[40]	✘	✘	✘	✓	✘	✓	✓	Monte Carlo	✘
[32]	✘	✘	✘	✓	✓	✘	✓	SO	✘
[41]	✓	✓	✘	✓	✓	✓	✘	actual user participation rate	✓
[42]	✓	✓	✘	✓	✘	✓	✓	Fuzzy method and SO	✓
[43]	✓	✓	✓	✓	✓	✘	✓	Latin Hypercube Sampling	✓
This paper	✓	✓	✓	✓	✓	✓	✓	Triangular Fuzzy	✓

(1) Most studies adopt a traditional single-price carbon trading mechanism, which offers limited incentives for enterprises to control carbon emissions [4–9];
(2) Current DR implementations either rely on simplified load flexibility classifications [37,38] or overemphasize peak shaving and operational cost reduction, thereby constraining the full exploitation of DR’s decarbonization potential;
(3) Despite extensive research on carbon trading and DR, their potential synergies remain underexplored;
(4) The system optimization model either adopts a deterministic model [35,36], or adopts traditional methods such as stochastic/robust optimization [30,34,37]. The former ignores uncertainty, while the latter is limited by ideal probability assumptions or conservative constraints, which is not conducive to scheduling optimization under complex scenarios in the real world.

To address these limitations, this paper proposes an optimal scheduling model that integrates DR and the carbon trading mechanism. First, based on the economic rationale of DR cost savings, a refined classification of flexible loads is developed, and compensation models are constructed for both shiftable and reducible loads. Second, a collaborative low-carbon mechanism is established by integrating DR on the load side and a carbon trading mechanism on the generation side. A reward–penalty laddered carbon trading mechanism is introduced to incentivize emissions control. To address system uncertainty, triangular fuzzy membership functions are used to characterize uncertain variables, and fuzzy chance constraints are transformed into deterministic constraints through equivalence class transformations. The model is solved using the commercial solver CPLEX, with the objective of minimizing the total cost of wind and thermal generation, DR compensation, and carbon trading. The proposed model’s economic and low-carbon performance is validated through case analysis and scenario comparison. As shown in Table 1, the proposed framework demonstrates broader capabilities than existing approaches. The main contributions of this paper are summarized as follows:

(1) Refined Demand Response Modeling: A flexible load classification is proposed based on the economic rationale of DR. Separate compensation models are constructed for shiftable and reducible loads.
(2) Reward and Penalty Laddered Carbon Trading Mechanism: Unlike the traditional single-price approach, a piecewise linear carbon pricing model with reward and penalty factors is introduced to further incentivize emissions reductions.
(3) Source-Load Synergistic Low Carbon Mechanism: The carbon trading mechanism on the supply side and the DR mechanism on the demand side are jointly incorporated into a day-ahead scheduling framework, enabling a coordinated strategy for low-carbon operation.
(4) Uncertainty Management: To address uncertainties in renewable generation and load forecasting, a triangular fuzzy approach is adopted. Fuzzy chance constraints are transformed into deterministic equivalents to facilitate efficient solution of the optimization problem.

The rest of this paper is organized as follows: Section 2 introduces the relevant principles as well as calculation models of the demand response mechanism and carbon trading mechanism. Section 3 provides solution methods to deal with the proposed models, including uncertainty treatment, objective function and constraints, condition transformation and solution process. Section 4 discusses the validity of the proposed model by taking a provincial grid as a simulation object. The main conclusions are presented in Section 5.

## 2 Methods

### 2.1 Demand response mechanism

**Fig 1:**
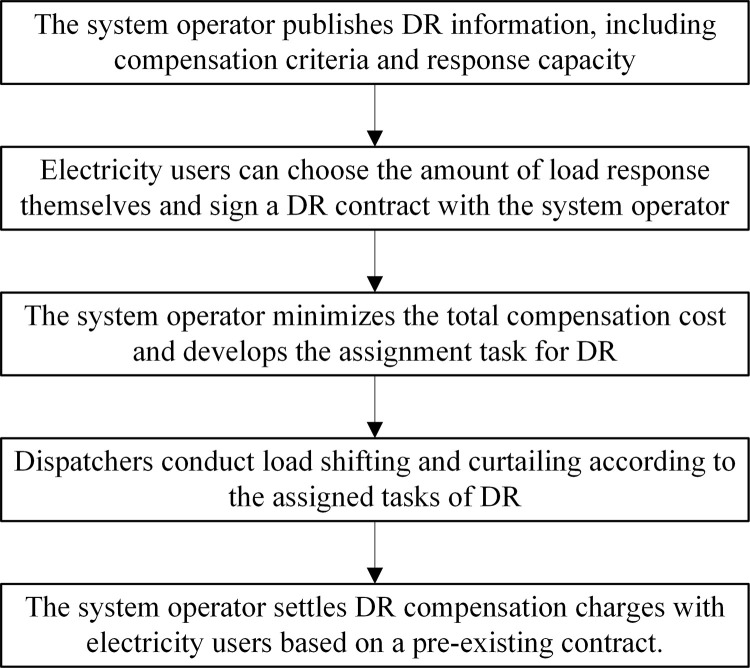
Incentivized DR implementation flow.

Demand response is usually classified into two categories: price-based DR and incentive-based DR. Price-based DR refers to the real-time tariffs that can be used by the dispatching organization to change the electricity consumption habits of the users when making the day-ahead scheduling plan. While incentive-based DR signs DR agreements with power users in advance and directly regulates the user load to realize quantitative flexible resource dispatch [44]. Compared with price-based DR, the amount of flexible load regulated by incentive-based DR is determined, without involving the formulation of electricity prices, more fully mobilizing the participation of power users. In additional, the implementation of incentive-based DR is simple, feasible and operational, as shown in the **Fig 1**.

#### 2.1.1 The economics of demand response.

**Fig 2:**
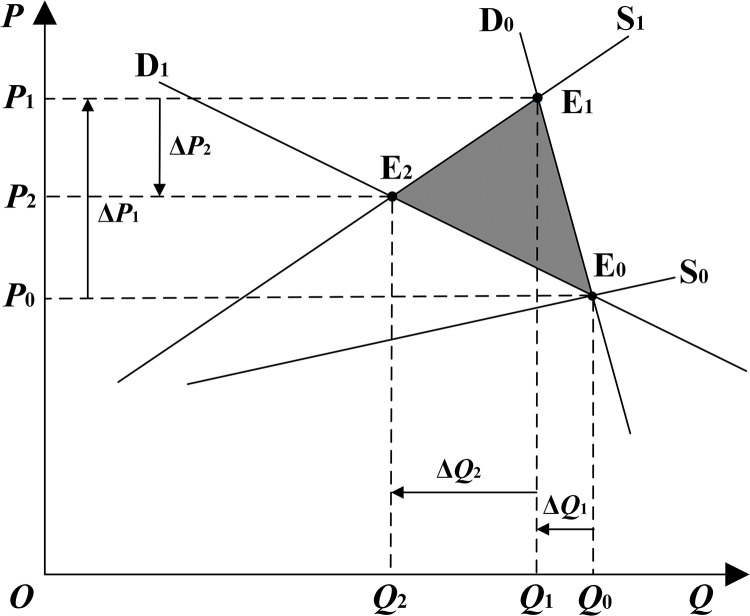
Costs saved by increased demand response.

According to the theory of market equilibrium and price elasticity in microeconomics [45], the effect of implementing DR on the system cost is analyzed, as illustrated in **Fig 2**. The P-axis and the Q-axis represent the price and the quantity of electricity in the market, respectively. The S-axis and the D-axis represent the supply curve and demand curve of the electricity market, respectively. Point E is the market equilibrium point.

(1) If DR is not implemented on the demand side, assuming that the elasticity of user demand is smal land insensitive to changes in market prices, the demand curve is approximately perpendicular to the *Q*-axis of D_0_. When the system generating capacity is abundant, the slope of the supply curve S_0_ is small and the supply price elasticity is large, manifesting as a flat curve. When the demand curve D_0_ and supply curve S_0_ intersect at the equilibrium point E_0_, the market supply and demand to achieve equilibrium, the market clearing price of *P*_0_, clearing the electricity amount of *Q*_0_. When the system generating capacity is in short supply, the slope of the supply curve increases and steepens from S_0_ to S_1_. Consequently, the demand curve *D*_0_ and the altered supply curve S_1_ intersect at the equilibrium point E_1_, causing the market price to rise to *P*_0_ + *Δ*P_1_. Due to the inelasticity of demand, the decrease in demand is small, denoted as Δ*Q*_1_.
(2) If the regulatory potential of flexible load resources is fully harnessed and the DR mechanism is implemented, the price elasticity of customer demand increases. This leads to greater sensitivity to market price fluctuations, prompting customers to adjust their electricity consumption in response to price signals or financial incentives. The demand curve shifts from D_0_ to D_1_, where it intersects with the supply curve S_1_ at the new equilibrium point E_2_. Consequently, the market price increase is reduced to Δ*P*_2_, and the decrease in demand rises to Δ*Q*_2_. When generating capacity is abundant, the market equilibrium is E_0_; further, when generating capacity is scarce, the market equilibrium is E1. And with the introduction of DR, the market equilibrium changes to E_2_. The shaded area in **Fig 2** illustrates that the introduction of DR into the electric power market generates cost savings.

#### 2.1.2 Demand response compensation model.

**Fig 3:**
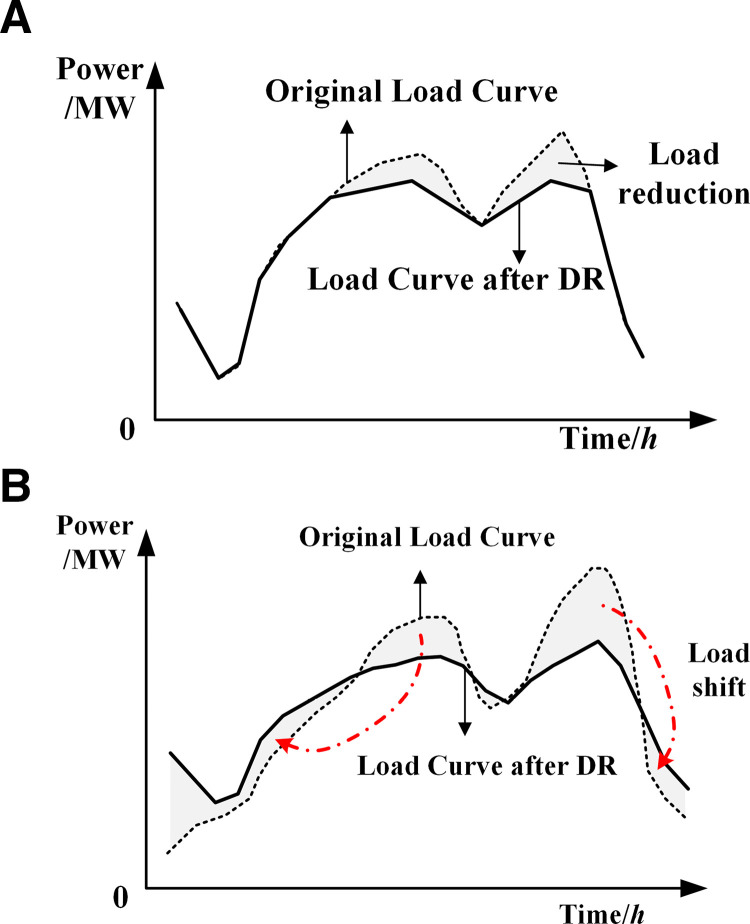
(a) Schematic diagram of shiftable load; (b) Schematic diagram of curtailable load.


Flexible loads are a crucial component of demand response resources. **Fig 3** illustrates the characteristics of shiftable and curtailable loads. The system’s electrical load $P_{d,t}$ comprises base load $P_{base,t}$, shiftable load $P_{shift,t}$ and curtailable load $P_{cut,t}$.

$$P_{d,t}=P_{base,t}+P_{shift,t}+P_{cut,t}$$ (1)

1 Shiftable load

Shiftable loads lack continuity constraints and are flexible in operation. The total load before and after the transfer must remain unchanged, as indicated in Equation (2).


$$\sum_{t=1}^{T}\left(L_{shift,t}-P_{shift,t}\right)=0$$ (2)

where $L_{shift,t}$ and $P_{shift,t}$ denote the shiftable load power in the pre- and post-optimization periods, respectively; *T* denotes the length of one operating cycle of the system.

The transfer time interval for the shiftable load is [$t_{tr-}$,$t_{tr+}$]. To prevent frequent starting and stopping of the equipment, constraints are placed on its minimum continuous operation time and power range.


$$\partial_{shift,t}P_{shift,min}\leq P_{shift,t}\leq \partial_{shift,t}P_{shift,max}$$ (3)

$$\sum_{T=t}^{t+T_{shift}^{\min}-1}\partial_{shift,t}\geq T_{shift,\min}(\partial_{shift,t}-\partial_{shift,t-1})$$ (4)

where $\partial_{shift,t}$ denotes the 0–1 variable, indicating the transfer state of the load at a certain time, with 1 denoting a shift and 0 denoting no shift; $P_{shift,max}$ and $P_{shift,min}$ represent the upper and lower limits of the shiftable power, respectively; $T_{shift}^{\min}$ denotes the minimum time of continuous operation.

2 Curtailable load

Curtailable loads ensure the safe and reliable operation of the power system by directly reducing power consumption. To ensure that load reduction does not affect customer satisfaction, constraints are placed on the upper and lower limits of reduction time and the frequency of reductions.


$$T_{cut,min}\leq \sum_{T=t}^{t+T_{kp}-1}\partial_{cut,t}\leq T_{cut,max}$$ (5)

$$\sum_{t=1}^{T}\partial_{cut,t}\leq N_{\max}$$ (6)

where $T_{kp}$ denotes the length of continuous cut; $T_{cut,max}$ and $T_{cut,min}$ represent the upper and lower limits of the length of continuous cut, respectively; $\partial_{cut,t}$ denotes the 0–1 variable, which indicates the state of load cut at a certain time period, with 1 denoting cut and 0 denoting no cut; $N_{max}$ denotes the maximum number of cuts.

3 Demand response compensation

The total compensation cost available to users for participating in incentive-based DR is composed of the DR volume and the unit compensation price, which can be divided into shiftable load and curtailable load compensation, calculated as shown in equations (7) to (9).


$$F_{shift}=F_{shift,cost}\sum_{t=1}^{T}P_{shift,t}$$ (7)

$$F_{cut}=F_{cut,cost}\sum_{t=1}^{T}P_{cut,t}$$ (8)

$$C_{1}=F_{shift}+F_{cut}$$ (9)

where $F_{shift}$ denotes the compensation cost after the shiftable load response; $F_{cut}$ denotes the compensation cost after the curtailable load response; $F_{shift,cost}$ denotes the compensation price per unit of shiftable load; $F_{cut,cost}$ denotes the compensation price per unit of curtailable load; $P_{cut,t}$ denotes the load power that can be cut at time period *t*; $C_{1}$ denotes the compensation cost received by users participating in demand response.

### 2.2 Source-Load synergistic low carbon mechanism

#### 2.2.1. Source-Load synergistic low carbon principle.

**Fig 4:**
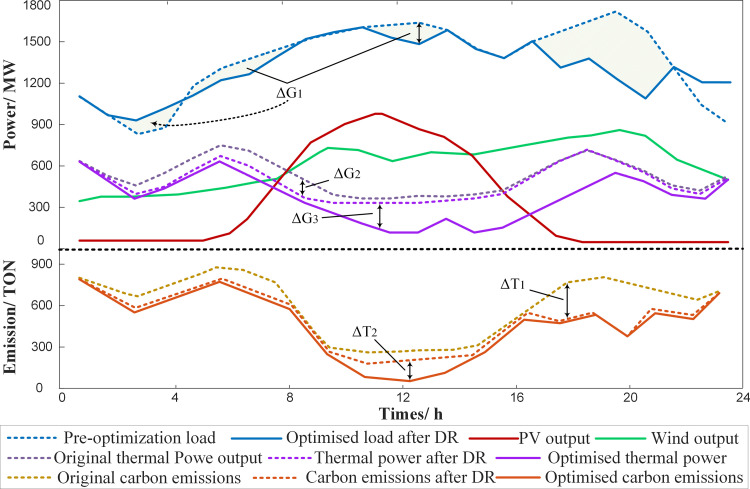
Source-load synergistic low carbon principle.

**Fig 4** illustrates the schematic of the source-load synergistic low-carbon principle, which incorporates DR and carbon trading mechanism. The low-carbon effect is primarily achieved through two key strategies: enhancing the integration of new energy and reducing carbon emissions. On the source side, the carbon trading mechanism directly reduces emissions by internalizing the cost of carbon and prioritizing the output of low-carbon generation units. On the load side, the implementation of incentive-based DR encourages users to adjust their electricity consumption behavior, achieving a “peak shaving and valley filling” effect. This approach smooths the load curve, alleviates the burden on thermal power generation during peak periods through load reduction or shifting, strengthens the coupling between load and renewable energy output, increases renewable energy utilization, and indirectly reduces carbon emissions.

As shown in **Fig 4**, the implementation of DR leads to load shifting and curtailment, resulting in changes in load denoted by ΔG₁ and ΔG₂. The optimized load curve becomes noticeably smoother. Simultaneously, the output of thermal power units during the 4:00–16:00 period decreases significantly. This reduction occurs because DR alleviates the pressure on thermal generation during peak hours by increasing the consumption of renewable energy. Effectively, low-carbon renewable energy output replaces high-emission thermal generation during peak periods, resulting in a carbon emissions reduction represented by ΔT₁. Furthermore, when the carbon trading mechanism is incorporated, thermal power units with higher carbon emission intensity reduce their output between 8:00 and 20:00 to lower carbon trading costs and emissions. This adjustment further enhances renewable energy utilization and results in an additional emissions reduction denoted as ΔT₂. In summary, DR on the load side, combined with the carbon trading mechanism on the source side, enables deeper utilization of flexibility resources. This coordination optimizes the dispatch of thermal and new energy units, lowers system-wide carbon emissions, and facilitates low-carbon economic dispatch.

#### 2.2.2. Laddered carbon trading mechanism.

The carbon trading mechanism achieves the goal of reducing carbon emissions by trading carbon emission allowances. Carbon emission quotas are allocated by government or regulatory authorities to incentive power generation enterprises to adhere to energy-saving and emission reduction policies [46].

1 Carbon Emission Quota System

This article uses the baseline method to determine the gratuitous carbon emission quota, considering that the allocation primarily includes conventional thermal power, purchased thermal power from external grids, wind power, and photovoltaic power. The system’s carbon emission quota can be expressed as


$$D_{q}=\sum_{t=1}^{T}\sum_{gi=1}^{N}\gamma_{gi}P_{gi,t}+\sum_{t=1}^{T}\gamma_{ip}P_{ip,t}+\sum_{t=1}^{T}\gamma_{re}(P_{w,t}+P_{pv,t})$$ (10)

where $D_{q}$ denotes the total carbon emission quota;$P_{gi,t}$ denotes the thermal power unit output in time period *t*; ${P_{ip,}}_{t}$ denotes the purchased electricity in time period *t*; ${P_{w,}}_{t}$ and ${P_{pv,}}_{t}$ denote *t*he wind power and PV output in time period *t*; N denotes the total number of thermal power uni*t*s; $\gamma_{gi}$ denotes the carbon emission quota of thermal power; $\gamma_{ip}$ denotes the carbon emission quota of purchased thermal power; $\gamma_{re}$ denotes the carbon emission quota of wind and photovoltaic power.

The actual total carbon emissions of the system mainly come from thermal power units and purchased thermal power, which can be calculated as follows


$$D_{a}=\sum_{t=1}^{T}\sum_{i=1}^{n}\eta_{gi}P_{gi,t}+\sum_{t=1}^{T}\eta_{ip}P_{ip,t}$$ (11)

where $D_{a}$ denotes the total actual carbon emissions of the system; $\eta_{gi}$ denotes the carbon emission intensity of thermal power units; $\eta_{gi}$ denotes the carbon emission intensity of purchased thermal power.

Then, the actual carbon trading volume $D_{m}$ in the grid is


$$D_{m}=D_{a}-D_{q}$$ (12)

2 Reward and punishment laddered carbon trading costs

When actual carbon emissions are below the allocated quotas, the surplus quota rights can be sold in the carbon trading market, generating revenue. Conversely, if emissions exceed the quotas, additional quota rights must be purchased. This paper builds on the traditional carbon trading mechanism by dividing actual carbon trading volumes into four intervals and introducing reward and punishment factors to create a laddered carbon trading cost model.


$$\begin{array}{c} \varepsilon =\left\{ \begin{array}{cc} (1+m)\varepsilon_{0} & \;D_{m}\leq -d\\ \varepsilon_{0} & \;-d\leq D_{m}\leq d\\ (1+k)\varepsilon_{0} & \;d\leq D_{m}\leq 2d\\ (1+2k)\varepsilon_{0} & \;2d\leq D_{m} \end{array}\right. \end{array}$$ (13)

$$\begin{array}{c} C_{2}=\{\begin{array}{cc} {}*20ll\varepsilon (D_{m}+d)-d\varepsilon_{0} & \;D_{m}\leq -d\\ D_{m}\varepsilon & \;-d\leq D_{m}\leq d\\ \varepsilon (D_{m}-d)+d\varepsilon_{0} & \;d\leq D_{m}\leq 2d\\ \varepsilon (D_{m}-2d)+d(1+k)\varepsilon_{0}+d\varepsilon_{0} & \;2d\leq D_{m} \end{array} \end{array}$$ (14)

where ε denotes the reward and penalty laddered carbon trading price; $\varepsilon_{0}$ denotes the initial carbon trading price; d denotes the unit increase interval of the actual carbon trading volume; m denotes the reward coefficient; k denotes the penalty coefficient; $C_{2}$ denotes the carbon trading cost.

**Fig 5:**
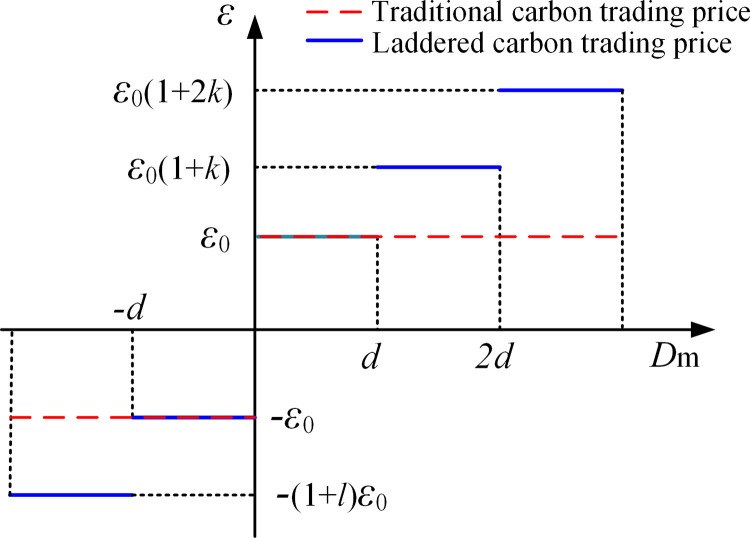
Schematic diagram of laddered carbon trading price.

In the carbon trading cost calculation model presented above, the relationship between the carbon trading price and trading volume is illustrated in **Fig 5**. The actual carbon trading volume is divided into four intervals. The horizontal axis represents the extent to which actual carbon emissions exceed the allocated carbon quota, while the vertical axis indicates the corresponding carbon trading price [47]. The specific trading price varies across intervals, and as the volume of excess emissions increases across higher intervals, the carbon trading price rises accordingly.

## 3. Solution methods

### 3.1 Source-Load uncertainty treatment

When large-scale renewable energy sources are integrated into the power grid, the associated prediction errors become significant and cannot be neglected. In the day-ahead power system dispatch model, both renewable energy output and load demand are treated as uncertain variables. In this study, the triangular fuzzy method is employed to model these uncertainties and the triangular affiliation parameter is represented as (15).


$$\left\{ \begin{array}{c} {\tilde{P}}_{w,t}=(P_{w,t}^{1},P_{w,t}^{2},P_{w,t}^{3})=P_{w,t}(\lambda_{1w},\lambda_{2w},\lambda_{3w})\\ {\tilde{P}}_{pv,t}=(P_{pv,t}^{1},P_{pv,t}^{2},P_{pv,t}^{3})=P_{pv,t}(\lambda_{1pv},\lambda_{2pv},\lambda_{3pv})\\ {\tilde{P}}_{d,t}=(P_{d,t}^{1},P_{d,t}^{2},P_{d,t}^{3})=P_{d,t}(\lambda_{1d},\lambda_{2d},\lambda_{3d}) \end{array}\right.$$ (15)

where ${\tilde{P}}_{w,t}$,${\tilde{P}}_{pv,t}$,${\tilde{P}}_{d,t}$ denote the fuzzy tabular form of wind power, PV output and electric load respectively; $P_{w,t}^{1}$ -$P_{w,t}^{3}$,$P_{pv,t}^{1}$ -$P_{pv,t}^{3}$,$P_{d,t}^{1}$ -$P_{d,t}^{3}$ denote the triangular affiliation parameter of the actual wind power output, the actual PV output and the actual electric load; ${P_{w,}}_{t}$,${P_{pv,}}_{t}$,${P_{d,}}_{t}$ denote the predicted value of the wind power, PV output and electric load; ${\lambda_{1}}_{w}$ -${\lambda_{3}}_{w}$,${\lambda_{1}}_{pv}$ -${\lambda_{3}}_{pv}$,${\lambda_{1}}_{d}$ -$\lambda_{3d}$ denote the ratio coefficients of the wind power, PV output and electric load, which is generally determined by the historical data.

### 3.2 Objective functions

Under the premise of satisfying the rotating standby, the article aims to minimize the system operating cost as the optimization objective.


$$\min F=C_{1}+C_{2}+C_{3}+C_{4}+C_{5}$$ (16)

where F denotes the total system operating cost; $C_{3}$ denotes the thermal power unit generation cost; $C_{4}$ denotes the wind power and PV operation and maintenance cost; $C_{5}$ denotes the cost of purchased electricity.

(1) Thermal Power Generation Cost. Thermal power output is stable and controllable. Its cost mainly includes coal consumption and the costs of starting and stopping the unit, calculated as follows:

$$C_{3}=\sum_{t=1}^{T}\sum_{i=1}^{N}u_{gi,t}(a_{i}P_{gi,t}^{2}+b_{i}P_{gi,t}+c_{i})+\sum_{t=1}^{T}\sum_{i=1}^{N}u_{gi,t}(1-u_{gi,t-1})S_{gi,t}$$ (17)

where $u_{gi,t}$ denotes the start-stop status of the thermal power unit; $a_{i}$,$b_{i}$,$c_{i}$ denote the coal cost coefficients of the thermal power unit, respectively; $S_{gi,t}$ denotes the start-stop cost of the thermal power unit.

(2) Wind and PV Operation and Maintenance Costs. Wind power and PV are renewable energy sources with no fuel consumption during power generation. Their costs mainly derive from operation and maintenance, calculated as follows:

$$C_{4}=\sum_{t=1}^{T}\left(\beta_{w}P_{w,t}+\beta_{pv}P_{pv,t}\right)$$ (18)

where $\beta_{w}$ and $\beta_{pv}$ denote the operation and maintenance cost coefficients of wind farms and photovoltaic plants, respectively.

(3) Cost of purchased electricity. The cost of purchased electricity is the multiplication of the purchased time share price and the amount of electricity purchased.

$$C_{5}=\sum_{t=1}^{T}P_{ip,t}p_{ip,t}$$ (19)

where ${p_{ip,}}_{t}$ denotes the time share price of purchased electricity.

### 3.3 Restrictions

(1) Power Balance Constraints. Since the system’s power balance and rotating standby constraints involve uncertain variables, the deterministic power balance formulation is no longer applicable. In this study, a fuzzy logic-based approach is adopted to address uncertainty by introducing fuzzy parameters. These parameters are used to construct an uncertainty set, thereby relaxing the deterministic power balance constraints into probabilistic constraints defined under a confidence level α. This ensures that the probability of satisfying the power balance constraints is not less than α. Therefore, the fuzzy chance constraint for power balance is shown in equation (20). The fuzzy chance constraint for rotating standby is shown in equation (21)


$$C_{r}\left\{{\tilde{P}}_{d,t}-{\tilde{P}}_{w,t}-{\tilde{P}}_{pv,t}-\sum_{gi=1}^{N}u_{gi,t}P_{gi,t}-P_{ip,t}=0\right\}\geq \alpha$$ (20)

$$C_{r}\left\{{\tilde{P}}_{d,t}-{\tilde{P}}_{w,t}-{\tilde{P}}_{pv,t}-\sum_{gi=1}^{N}u_{gi,t}P_{gi,t,\max}-P_{ip,t}\leq 0\right\}\geq \alpha$$ (21)

where $C_{r}\{\cdot \}$ denotes the confidence level of seeking event {·}; α denotes the confidence level of plausibility;$P_{gi,i,\max}$ denotes the maximum output of thermal power units during the time period.

(2) Thermal power unit related constraints

Thermal unit output constraints:


$$P_{gi,t,\min}\leq P_{gi,t}\leq P_{gi,t,\max}$$ (22)

where $P_{gi,t,\min}$ denotes the minimum thermal unit output during the time period

Thermal power unit climbing constraint:


$$-R_{gi,down}\Delta t\leq (P_{gi,t}-P_{gi,t-1})\leq R_{gi,up}\Delta t$$ (23)

where ${R_{gi}}_{,down}$ and ${R_{gi}}_{,up}$ denote the maximum downward and upward climbing rate of the thermal power unit, respectively.

Minimum start-stop time constraint for thermal power unit:


$$\left\{ \begin{array}{c} (u_{gi,t-1}-u_{gi,t})(T_{gi,t-1}-T_{gi,on})\geq 0\{u_{gi,t}\}-u_{gi,t-1})(-T_{gi,t-1}-T_{gi,off})\geq 0 \end{array}\right.$$ (24)

where $T_{gi,on}$ and $T_{gi,off}$ denote the maximum continuous start-up time and maximum continuous shutdown time of the thermal power unit, respectively.


$$\begin{array}{c} 0\leq P_{w,t}\leq P_{w,t,max}\\ 0\leq P_{pv,t}\leq P_{pv,t,max} \end{array}$$ (25)

where $P_{w,t,max}$, $P_{pv,t,max}$ denotes the upper limit of the output of wind farms and PV power plants during the time period, respectively.

### 3.4 Model solution

Due to the optimal scheduling model incorporates fuzzy opportunity constraints, it falls under the category of uncertainty optimization models. The key to solving such problems lies in transforming the opportunity constraints into their deterministic equivalents. Two primary methods are commonly used: the clear equivalence class transformation and stochastic simulation techniques. The clear equivalence class transformation method separates the fuzzy parameters in the constraints from the decision variables, allowing the problem to be solved using conventional optimization methods after transformation. In contrast, stochastic simulation techniques involve numerous iterations, are computationally intensive, and often yield less precise results. Therefore, this study adopts the clear equivalence class transformation method to address the fuzzy chance constraint problem. Referring to References [20,48,49], after applying this transformation, the power balance and spinning reserve constraints are converted into equations (26) and (27).


$$(2-2\alpha )(P_{d,t}^{2}-P_{w,t}^{2}-P_{pv,t}^{2})+(2\alpha -1)(P_{d,t}^{3}-P_{w,t}^{1}-P_{pv,t}^{1})-\sum_{gi=1}^{N}u_{gi,t}P_{gi,t}-P_{ip,t}=0$$ (26)

$$(2-2\alpha )(P_{d,t}^{2}-P_{w,t}^{2}-P_{pv,t}^{2})+(2\alpha -1)(P_{d,t}^{3}-P_{w,t}^{1}-P_{pv,t}^{1})-\sum_{gi=1}^{N}u_{gi,t}P_{gi,t,\max}-P_{ip,t}\leq 0$$ (27)

where $P_{d,t}^{2}$ and $P_{d,t}^{3}$ denote the affiliation parameter of load; $P_{w,t}^{2}$ and $P_{w,t}^{1}$ denote the affiliation parameter of wind power; $P_{pv,t}^{2}$ and $P_{pv,t}^{1}$ denote the affiliation parameter of PV.

**Fig 6:**
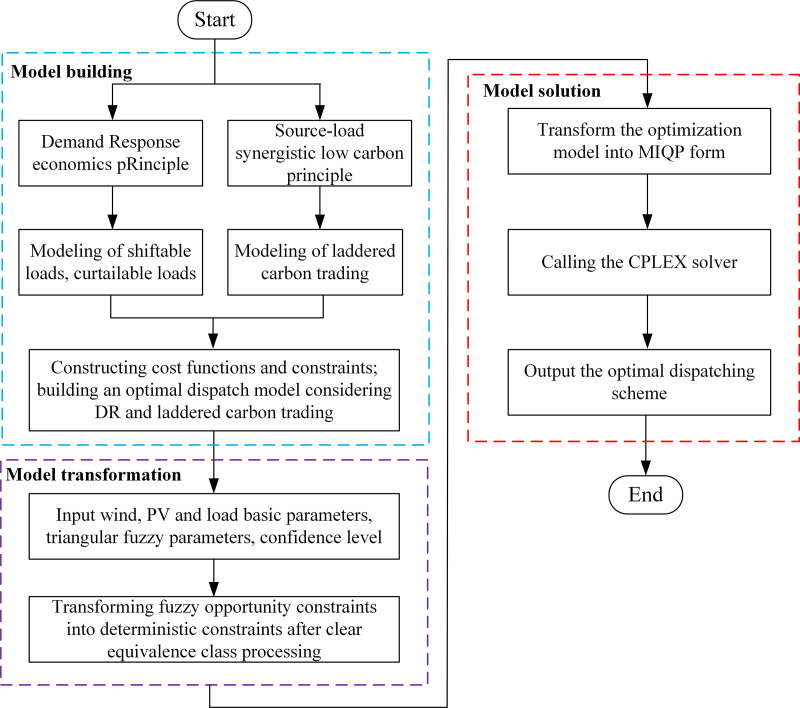
Flowchart of the solving process.

After converting the fuzzy constraints, the optimization model follows the same solution process as a conventional deterministic model. It is formulated as a Mixed-Integer quadratic programming (MIQP) problem. In this study, the model is solved using the commercial solver CPLEX, accessed via YALMIP within the MATLAB R2017b environment. The specific solution procedure is illustrated in **Fig 6**.

## 4. Simulation results and discussion

### 4.1. Basic settings

**Fig 7:**
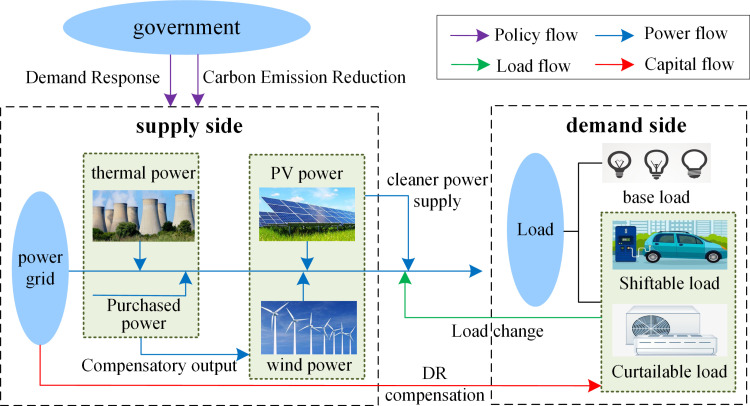
Provincial power grid integrated energy system.

This study investigates the integrated energy system of a regional provincial power grid, which comprises five conventional thermal power units, one wind farm, and one PV power plant. The structural layout of the system is illustrated in **Fig 7**. On the energy supply side, energy is provided by conventional thermal power, wind power, PV generation, and purchased electricity, ensuring sufficient supply to meet user demand. On the demand side, power users adjust their energy consumption in response to incentive-based DR programs with subsidized pricing. This approach reduces users’ energy costs while maintaining the balance between supply and demand, thereby supporting the safe and stable operation of the system.

**Fig 8:**
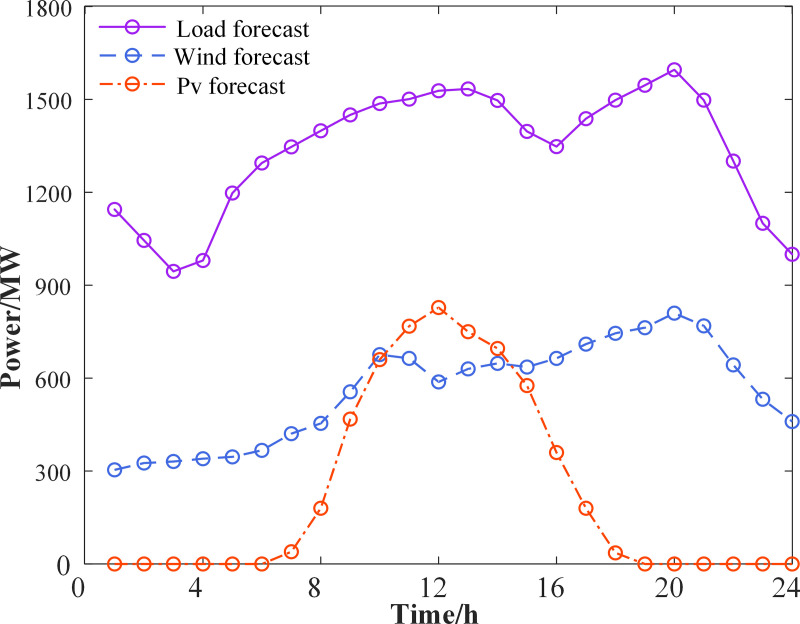
Forecasting curves of wind power 、PV output and load.

In this calculation example, the operating parameters of conventional thermal power units are provided in **Table 2**, sourced from the literature [50]. The carbon emission quotas and carbon emission intensity values are presented in **Table 3**. Additional parameters are listed in **Table 4**. The wind and PV output, as well as the load forecast curves, are shown in **Fig 8**. The values for the triangular fuzzy affiliation parameters are given in **Table 5** [38]. Flexible load parameters are provided in **Table 6** [54]. The initial carbon trading price ε is set at 150 yuan/t, with a unit increment interval d of 60t. The price reward factor l is 0.3, and the penalty factor k is 0.25 [43,55].

**Table 2:** Thermal power units operating parameters.

Unit	Maximum output/MW	Minimum output/MW	Climbing rate/MW	$T_{gi,on}$ /$T_{gi,off}$ /h	Coal-fired cost factor			$S_{gi}$ /(CNY/times)
					$a_{i}$ /(CNY/MW2)	$b_{i}$ /(CNY/MW)	$c_{i}$ /(CNY)	
G1	600	180	66	8	0.00169	0.27601	11.46196	30000
G2	300	150	35	6	0.00169	0.27601	11.46196	20000
G3	300	150	35	6	0.00169	0.27601	11.46196	20000
G4	200	100	25	3	0.01307	0.23222	16.00726	10000
G5	200	100	25	3	0.11241	0.28730	4.07362	10000

**Table 3:** Carbon emission allowances and Carbon emission intensity of thermal power units.

Unit	$\gamma_{gi}$ /(T/MWh)	Refs.	$\eta_{gi}$ /(T/MWh)	Refs.
G1	0.7	[51]	0.8	[52]
G2	0.798	[46]	0.905	[51]
G3	0.798	[46]	0.905	[51]
G4	0.728	[43]	0.97	[20]
G5	0.728	[43]	1.08	[20]

**Table 4:** Related parameter values.

Parameters	Express	Retrieve a Value	Refs.
Wind/PV operating cost factor	$\beta_{w}$ /$\beta_{pv}$	180/150 CNY/ (MW·h)	[20]
Carbon emission allowances for purchased thermal power	$\gamma_{ip}$	0.7 KG/(KW·h)	[51]
Carbon intensity for purchased thermal power	$\eta_{ip}$	0.8 KG/(KW·h)	[52]
Carbon emission allowances for Wind and PV	$\gamma_{re}$	0.078 KG/(KW·h)	[46]
Acceptable confidence levels for grid dispatch	α	0.90	[20]
Time share tariffs for purchased electricity	Peak, valley and trough tariffs	0.377/0.291/0.174 CNY/(KW·h)	[53]

**Table 5:** Parameter of triangular fuzzy membership parameter.

Fuzzy Parameters	$\lambda_{1}$	$\lambda_{2}$	$\lambda_{3}$
$\lambda_{w}$	0.9	1	1.1
$\lambda_{pv}$	_0.95_	1	1.05
$\lambda_{d}$	0.95	1	1.05

**Table 6:** Flexible load parameter.

Load	$T_{shift,\min}$ /H	$P_{shift,min}\sim P_{shift,max}$ /MW	[$t_{tr-}$, $t_{tr+}$].	$F_{shift,cost}$ / CNY/KWH
$P_{shift,t}$	2	50 ~ 300	04:00 ~ 18:00	0.1
Load	$T_{cut,min}$ /H	$T_{cut,max}$ /H	$N_{\max}$ /times	$F_{cut,cost}$ / CNY/KWH
$P_{cut,t}$	2	5	8	0.35

### 4.2. Optimized results

**Fig 9:**
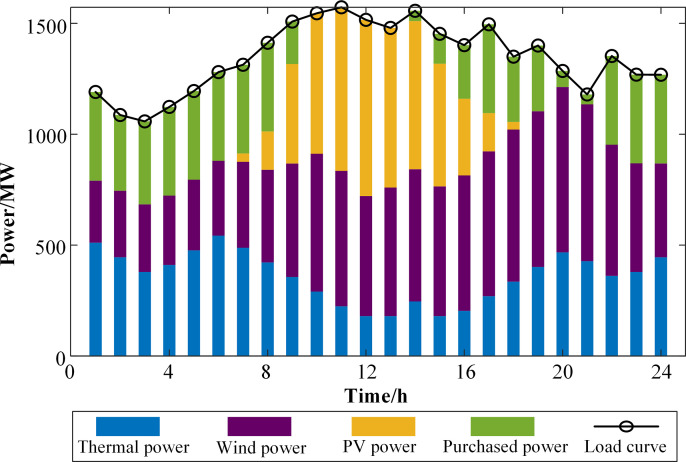
System power balance.

At a confidence level of 0.90, considering the uncertainties of new energy output and load forecasting, as well as carbon emission cost, the comprehensive system cost is 8.7976 million yuan. The actual carbon trading volume participating in the market is 10 tons sold, resulting in a carbon trading revenue of 0.15 million yuan. At this point, the total carbon emissions amount to 11,728.84 tons. Wind and PV power generation are fully utilized, and only the G1 thermal power unit, which has a lower carbon emission intensity, participates in power generation. Simultaneously, purchased power provides rotational reserves to address uncertainty. The results of the system’s optimal dispatching are illustrated in **Fig 9**.

**Fig 10:**
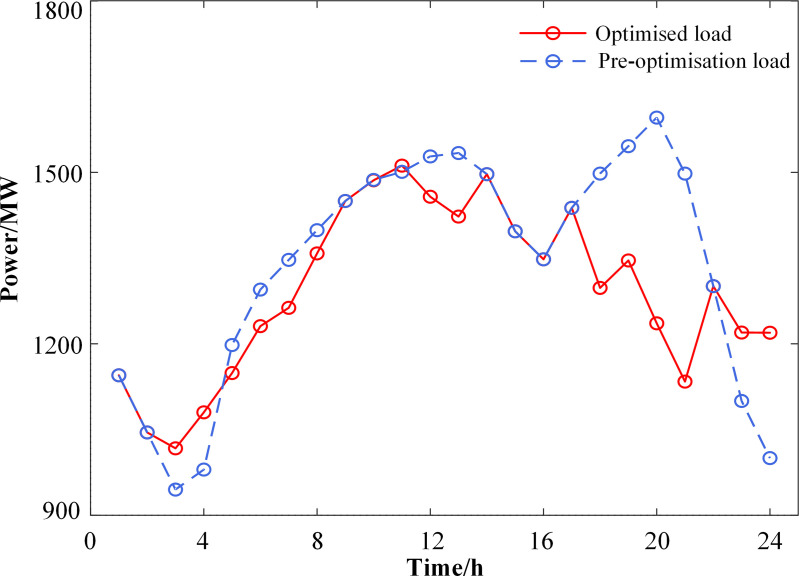
Comparison of load before and after demand response.

**Fig 10** illustrates the changes in load before and after optimization. It is evident that the maximum peak-to-valley difference of the load curve before optimization is 651 MW. In contrast, the load curve after optimization exhibits a noticeably smoother profile, with the maximum peak-to-valley difference reduced by 181 MW. This reduction indicates that the model presented in this paper facilitates peak shaving and valley filling.

**Fig 11:**
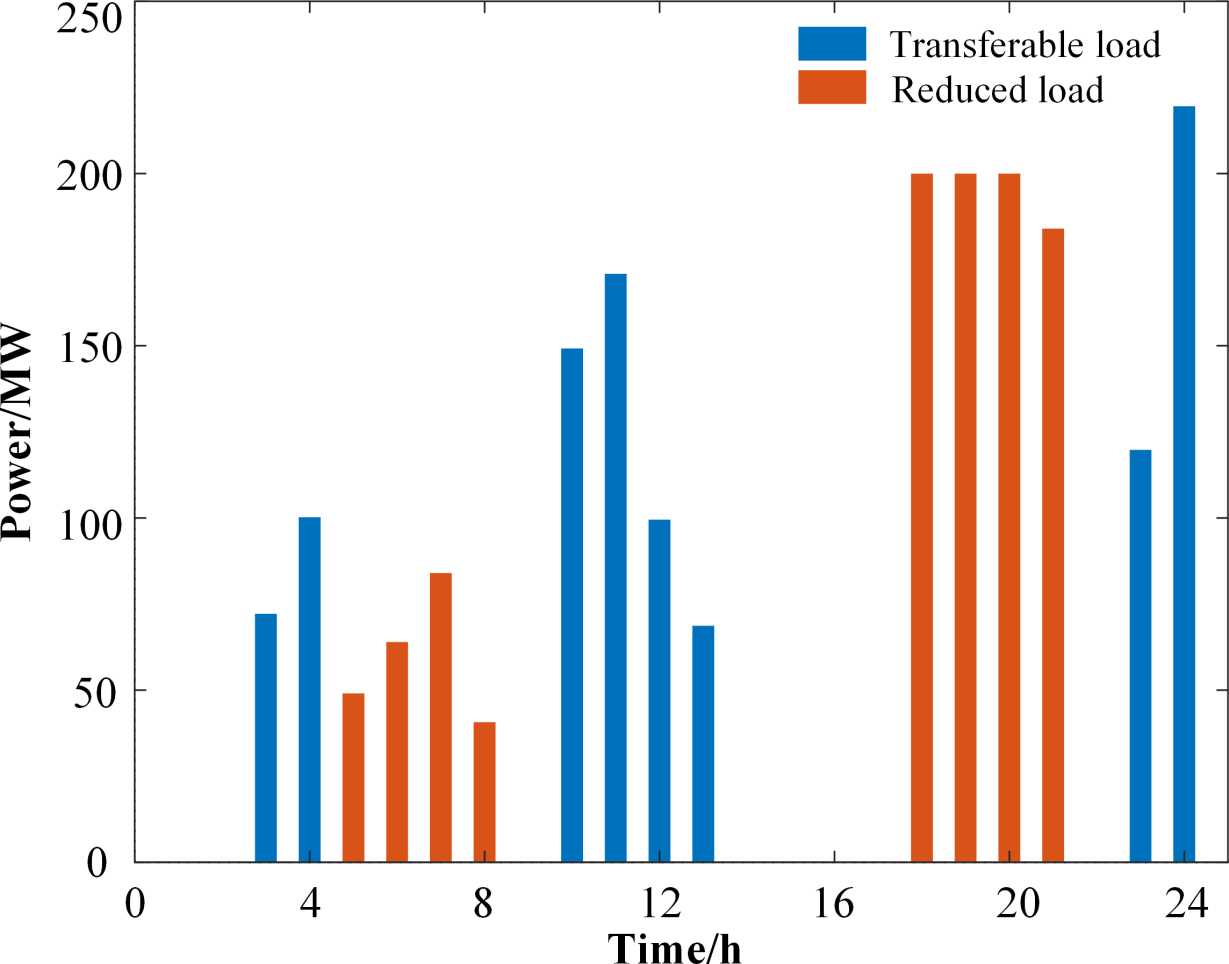
Response volume of flexible loads.

**Fig 11** illustrates the response of the flexible load. Particularly, during the time intervals from 17:00–21:00 and from 05:00–08:00, the optimized load is significantly reduced. This reduction is attributed to the strong anti-peaking characteristics of new energy output during peak electricity consumption periods. while purchased time-sharing tariffs are high, resulting in expensive energy supply costs. Therefore, appropriately reducing the peak load during these periods can significantly decrease electricity consumption costs. During the time intervals from 23:00–04:00 and from 10:00–13:00, a shift in the optimized load occurs. This shift is due to these hours being considered as the trough period of electricity consumption, coupled with sufficient wind power output and low purchased time-sharing tariff, thus reducing cost expenditure.

### 4.3. Comparative analysis of different scenarios

To evaluate the effectiveness of incorporating the incentive-based DR mechanism and the laddered carbon trading mechanism in system optimal scheduling, four simulation scenarios are established in this study, as shown in Table 7.

**Table 7:** Different scenario settings.

scenarios	Reward and punishment ladder carbon trading	Demand response
1	×	×
2	✓	×
3	×	✓
4	✓	✓

**Fig 12:**
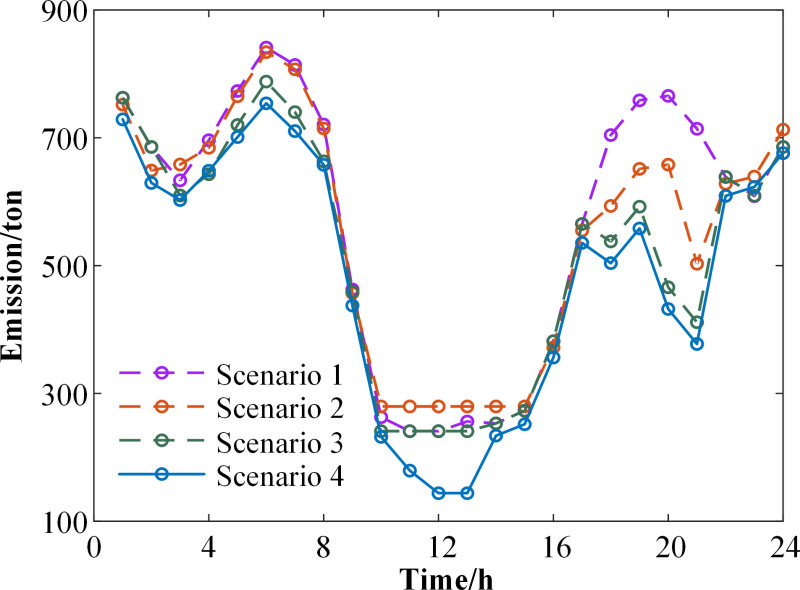
Carbon emissions for four scenarios.

**Fig 12** presents a comparison of carbon emissions across the four scenarios. Scenario 4 exhibits a significant reduction in carbon emissions compared to Scenarios 1, 2, and 3. Furthermore, Scenario 4 fully consumes the wind and PV, leading to an enhanced level of new energy consumption. Considering demand response and carbon trading simultaneously proves beneficial in achieving the objectives of carbon emission reduction and energy structure transformation.

Under scenarios with consistent parameters, the costs and carbon emissions for the four cases are presented in **Table 8**. Comparing Scenario 2 with Scenario 1, the implementation of the laddered carbon trading mechanism in Scenario 2 increases the output of new energy units to reduce carbon emissions. However, this implementation results in higher purchased energy costs, leading to a 1.89% increase in the total system cost. Despite the cost increase, Scenario 2 achieves a significant 3.11% reduction in carbon emissions. Additionally, the new energy curtailment rate decreases from 5.11% to 2.6%. These findings suggest that the laddered carbon trading mechanism not only effectively reduces system carbon emissions but also enhances the utilization of new energy sources.

**Table 8:** Cost comparison for four scenarios.

Costs	Scenario 1	Scenario 2	Scenario 3	Scenario 4
Integrated costs /ten thousand CNY	870.35	886.85	868.22	879.76
Energy consumption costs /ten thousand CNY	870.35	883.31	821.10	834.15
Carbon trading costs /ten thousand CNY	/	3.54	/	-0.15
DR compensation /ten thousand CNY	/	/	47.12	45.76
Carbon emission/ TON	13741.70	13314.50	12452.21	11728.84
Wind/PV abandonment rate	5.11%	2.60%	1.15%	0

Comparing Scenario 3 with Scenario 1, Scenario 3 reduces the peak supply pressure on thermal power units by shifting and curtailing flexible loads through the introduction of the DR mechanism. This alleviation of peak pressure leads to a reduction in the system’s energy purchase cost. Specifically, the purchased energy cost in Scenario 3 decreases by 5.65% compared to Scenario 1. Although the incentive-based DR introduces additional compensation costs due to subsidies provided for demand response participation, it significantly enhances the utilization of new energy, resulting in a 9.38% reduction in carbon emissions and a decrease in the new energy curtailment rate from 5.11% to 1.15%. These outcomes indicate that the DR mechanism indirectly reduces system carbon emissions by increasing the integration of renewable energy.

Comparing Scenario 4 with Scenarios 2 and 3, Scenario 4 integrates both the DR mechanism and the laddered carbon trading mechanism.Compared to Scenario 2, the implementation of DR in Scenario 4 achieves “peak shaving and valley filling” reduces peak load pressure on thermal power units, and further enhances the efficient utilization of renewable energy by accommodating fluctuations in the output of intermittent new energy sources. Specifically, the total system cost is reduced by 0.8%, and carbon emissions are reduced by 11.9%.Compared to Scenario 3, Scenario 4 adopts a laddered carbon trading mechanism. Since system carbon emissions fall below the allocated free allowances, surplus allowances can be sold, generating a carbon trading revenue of 0.15 million yuan. Although Scenario 4 incurs higher energy purchase costs than Scenario 3, carbon emissions are reduced by 5.81%, and all wind and PV power is fully utilized. In summary, the model proposed in this study effectively achieves emission reduction targets while maintaining system economic efficiency.

**Fig 13:**
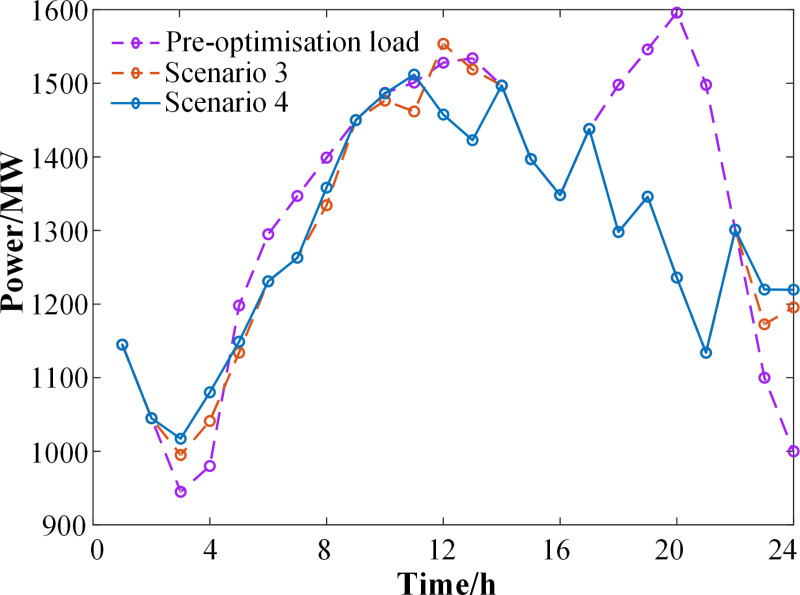
Load curve comparison.

**Fig 13** compares the electrical load profiles of the pre-optimization load, Scenario 3, and Scenario 4. As shown in **Fig 13**, the wind power output is higher during the time from 0:00–5:00, while the original load demand is lower, resulting in significant wind power abandonment. The introduction of demand response shifts and cut the load curve to varying extents. Scenario 4 demonstrates a smoother load curve compared to Scenario 3, achieving the most effective peak shaving and valley filling.

**Fig 14:**
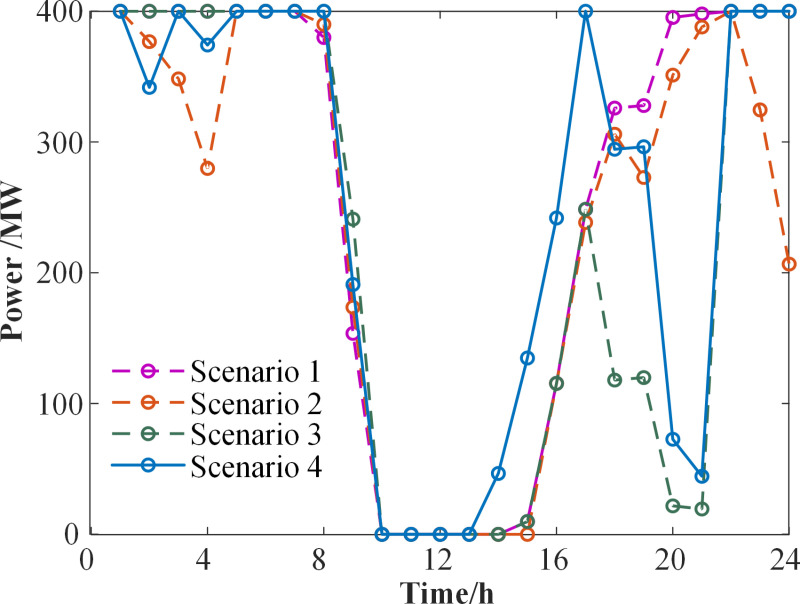
Comparison of purchased power output.

**Fig 14** depicts the power output of purchased electricity across various scenarios. When the share of purchased thermal power is 100%, the inclusion of the carbon trading mechanism results in higher carbon trading costs for the purchased thermal power. Consequently, Scenario 2 shows a 9.17% decrease in the power output of purchased electricity compared to Scenario 1. Additionally, when comparing Scenarios 2 and 3, the peak load is both shifted and reduced to some extent following the introduction of DR. This leads to a significant decrease in the purchased power output between 16:00 and 20:00. In Scenario 4, which takes into account the costs of purchased power, carbon trading, and DR compensation, the purchased power increases between 13:00 and 17:00.

### 4.4 Comparative analysis of different uncertainty treatments

To further compare the optimization results of different uncertainty modeling approaches, this study evaluates the triangular fuzzy method alongside stochastic optimization (SO) and robust optimization (RO) models, based on Scenario 4. Employing the control variable method, the uncertainty modeling approach is treated as the experimental variable, while all other conditions (e.g., operating environment, algorithm, and example parameter settings) are held constant. The robustness coefficient is set to 0.1, and a boxed uncertainty set is adopted. For the stochastic optimization model, the number of scenarios is set to 8 [56]. The optimization results for each model are presented in **Table 9**.

**Table 9:** Results comparison for different uncertainty treatments.

Costs	Triangular fuzzy	SO	RO
Integrated costs /ten thousand CNY	879.76	792.78	954.8
Energy consumption costs /ten thousand CNY	834.15	726.13	891.34
DR compensation /ten thousand CNY	45.76	39.65	32.13
Carbon emission/ TON	11728.84	12796.46	12377.53
Wind/PV abandonment rate	0	5.34%	1.12%
Calculation time/S	611.7	2258.2	632.8

Among comparing the three optimization methods, SO, which minimizes the expected cost based on the probability distribution of multiple scenarios, demonstrates the most favorable economic performance, achieving the lowest total cost of 7.928 million yuan. In contrast, RO, which makes decisions based on the worst-case scenario, adopts a more conservative strategy and results in the highest total cost of 9.548 million yuan. The triangular fuzzy optimization method, by selecting appropriate confidence levels, effectively balances system reliability and economic efficiency, with a total cost of 8.798 million yuan—positioned between those of SO and RO.

In addition, SO requires the longest computation time, as it necessitates a large number of scenarios to ensure the accuracy of the optimization results. In contrast, RO considers only boundary scenarios with a low probability of occurrence, making it less computationally intensive than SO. However, the resulting strategies tend to be more conservative. The triangular fuzzy method, which can utilize an expert system for the affiliation function in the absence of historical data, offers a simpler computational process and shorter computation time compared to RO. Furthermore, based on the results of carbon emissions and the new energy curtailment rate, the triangular fuzzy optimization method proposed in this paper achieves full utilization of new energy and yields the lowest carbon emissions among the compared models. In conclusion, the triangular fuzzy method demonstrates superior robustness and optimization performance compared to RO and SO, indicating that the proposed approach effectively enhances the dispatch strategy’s ability to handle uncertainty.

### 4.5. Sensitivity analysis

#### 4.5.1. Carbon trading price.

**Fig 15:**
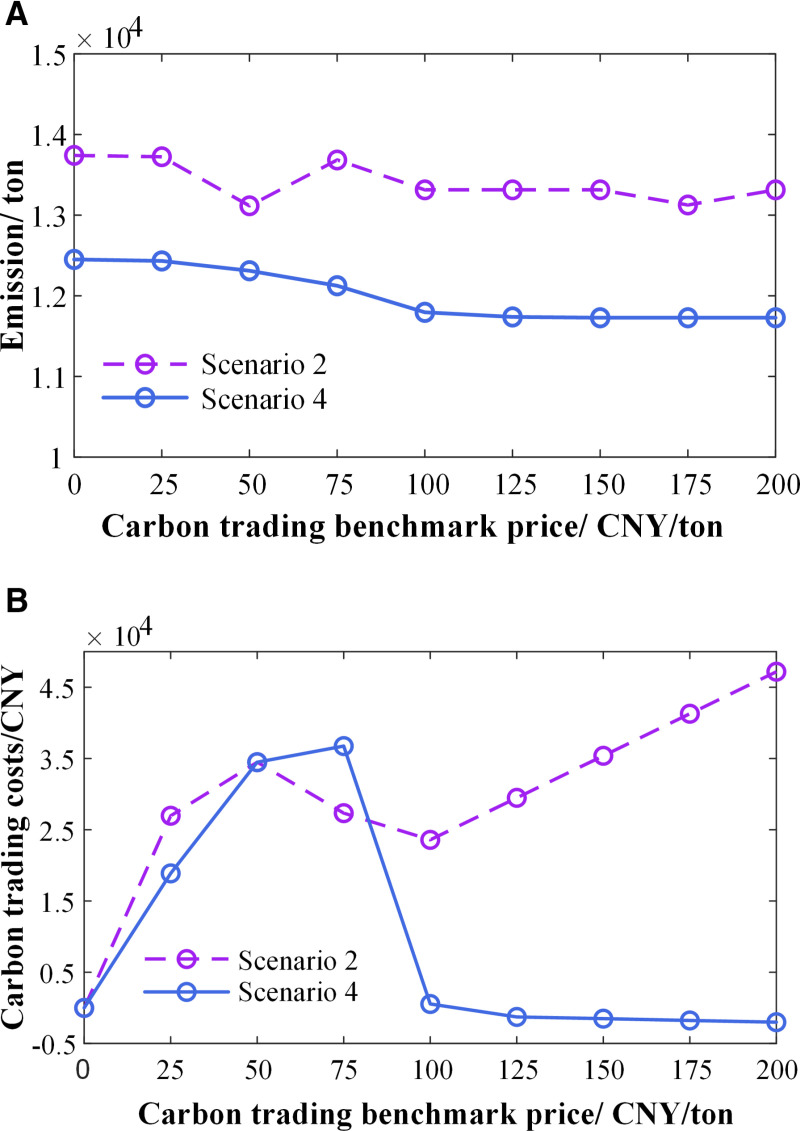
(a) The impact of carbon trading prices on carbon emissions; (b) The impact of carbon trading prices on carbon trading costs.

Changes in the carbon trading price will affect the carbon trading cost, which in turn will alter the system’s configuration results. Both Scenario 2 and Scenario 4 involve carbon trading mechanisms, and the trends in carbon emissions and carbon trading costs with varying carbon trading prices are compared between the two, as illustrated in **Fig 15**. It is evident that carbon emissions in both scenarios show a decreasing trend, but the emissions in Scenario 4 are significantly lower and decrease at a faster rate than those in Scenario 2. Additionally, as the carbon trading price increases, the carbon trading cost in Scenario 2 first rises, then falls, and subsequently increases steadily. The carbon trading cost in Scenario 4 increases and then decreases, and when the carbon trading price exceeds 100 CNY/ ton, the cost in Scenario 4 is considerably lower than in Scenario 2. This fully demonstrates that the model proposed in this paper effectively balances economic and low-carbon benefits, aligning with China’s national policy of promoting renewable energy development, energy conservation, and emission reduction.

#### 4.5.2. Confidence level.

**Fig 16:**
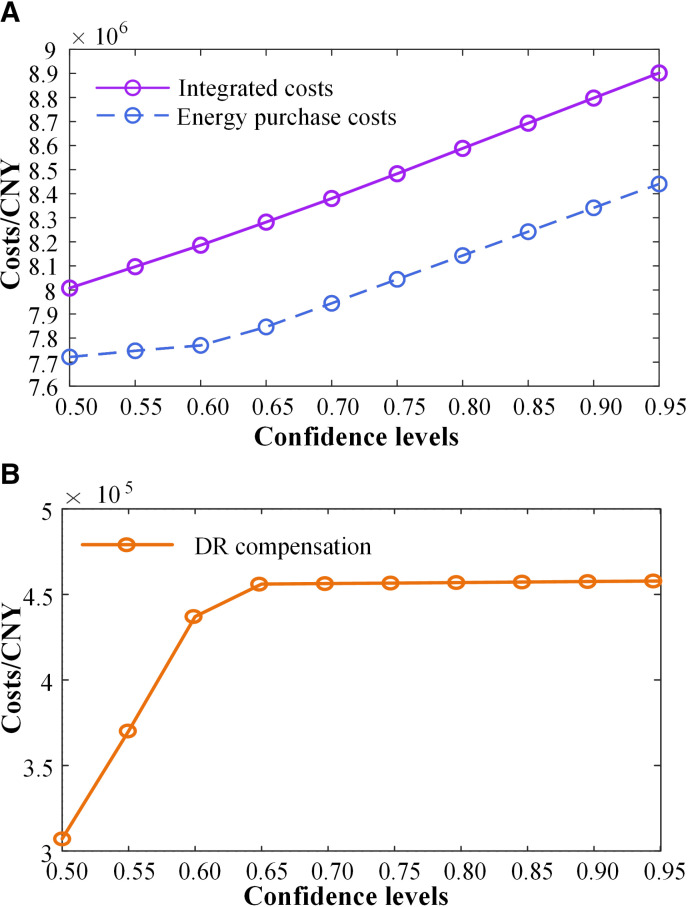
(a) The impact of different levels of confidence on integrated and energy consumption costs; (b) The effect of different confidence levels on DR compensation.

To further study the impact of uncertainty on system scheduling results, we analyze the changes in system integrated cost, energy purchase cost, and DR compensation cost under different confidence levels, as shown in Fig 16.We can see that increasing the confidence level α results in higher integrated costs, energy purchase costs, and DR compensation costs. This is because the confidence level represents the reliability of system operation, and a higher α requires greater operational reliability. To mitigate risks caused by source-load uncertainty, thermal power units and purchased power must increase output to maintain system power balance, significantly raising energy purchase costs. Additionally, the DR compensation mechanism reduces the peak-to-valley difference in the load curve and improves the spatial-temporal coupling between energy supply and usage. However, considering the constraints of power consumption satisfaction, the regulation of flexible loads is limited, leading to the compensation cost first increasing and then stabilizing.

## 5 Conclusion

To achieve the goals of supply security and low-carbon environmental protection, this paper proposes an optimization model that integrates DR compensation and a carbon trading mechanism under conditions of source-load uncertainty. On the demand side, the DR mechanism is introduced to fully explore the regulation potential of flexible load resources, focusing on the modeling and analysis of shiftable and curtailable loads. On the power generation side, the model promotes the coordination and complementarity of different energy sources through the rational arrangement of thermal power units, new energy generating units, and purchased power output to ensure stable system operation. The system’s source-load uncertainty is addressed using a triangular fuzzy approach, with the confidence level used to constrain operational reliability. Additionally, a laddered carbon trading mechanism is introduced to increase wind power and PV consumption, aiming to save energy and reduce emissions. Finally, the model minimizes DR compensation cost, carbon trading cost, and system operation cost to obtain the optimal dispatching scheme. The key findings are as follows:

(1) The DR compensation mechanism fully utilizes the regulation potential of demand-side flexible resources. By involving shiftable and curtailable loads, we can rationally adjust the electric load curve and reduce the peak-to-valley difference. This improves new energy consumption levels, reduces carbon emissions and energy purchase costs, and effectively balances economic and environmental benefits.
(2) To ensure the system’s low-carbon nature, we verify that the proposed model more stringently controls carbon emissions by comparing clearing results with and without the carbon trading mechanism. We analyze the impact of carbon trading prices on carbon emissions and costs, concluding that carbon emissions gradually decrease as the carbon trading price increases, while the total carbon trading cost initially rises rapidly, then decreases rapidly, and eventually shows a steady downward trend.
(3) To address system uncertainty, the triangular fuzzy approach can be employed. A comparison of the optimization results from the triangular fuzzy, SO and RO methods indicates that the triangular fuzzy approach offers superior robustness and economic performance. Meanwhile, the impact of varying confidence levels on system costs is analyzed. The results show that as the confidence level increases, the overall system cost also rises. Therefore, selecting an appropriate confidence level is essential to achieving a balance between system risk and economic efficiency.

This paper examines a low-carbon economic dispatch model that integrates DR and a carbon trading mechanism under source-load uncertainty. However, some limitations persist in this study. First, the study exclusively addresses incentive-based DR measures for electric loads. Expanding the model to incorporate heat and cooling loads, as well as price-based demand response strategies, is a potential direction for further research. Additionally, new low-carbon strategies may emerge during the power dispatch process. Future research could explore various pathways and measures to achieve energy savings and emission reductions from multiple perspectives. Furthermore, energy storage has been demonstrated to effectively improve the level of renewable energy consumption. Future research could incorporate energy storage systems to develop optimal scheduling strategies.

## Supporting information

S1 Data (XLS)
